# Supplementary material for: Modeling glioblastoma heterogeneity as a dynamic network of cell states
Source: Mol Syst Biol. 2021 Sep 16;17(9):e10105. doi: 10.15252/msb.202010105 (PMC8444284; doi:10.15252/msb.202010105)
Supplement: Supplementary file 6 — Source Data for Figure 5 [file MSB-17-e10105-s004.zip › Figure5A_sourcedata/GSEA_3017/nosigdb_stateD.GseaPreranked.1622795010397/RICHARDS_NOWAKOWSKI_SCIENCE_2017_IPC_NEN1_UPREG_1.html]

Details for gene set RICHARDS\_NOWAKOWSKI\_SCIENCE\_2017\_IPC\_NEN1\_UPREG\_1[GSEA]

|  || Dataset | state23017 |
| Phenotype | NoPhenotypeAvailable |
| Upregulated in class | na\_pos |
| GeneSet | RICHARDS\_NOWAKOWSKI\_SCIENCE\_2017\_IPC\_NEN1\_UPREG\_1 |
| Enrichment Score (ES) | 0.13151592 |
| Normalized Enrichment Score (NES) | 0.86075264 |
| Nominal p-value | 0.6666667 |
| FDR q-value | 0.7619251 |
| FWER p-Value | 0.997 |
Table: GSEA Results Summary

  

Fig 1: Enrichment plot: RICHARDS\_NOWAKOWSKI\_SCIENCE\_2017\_IPC\_NEN1\_UPREG\_1      
 Profile of the Running ES Score & Positions of GeneSet Members on the Rank Ordered List

  

| PROBE | GENE SYMBOL | GENE\_TITLE | RANK IN GENE LIST | RANK METRIC SCORE | RUNNING ES | CORE ENRICHMENT || 1 | SOX11 |  |  | 6 | 0.578 | 0.0456 | Yes |
| 2 | MEX3A |  |  | 30 | 0.402 | 0.0628 | Yes |
| 3 | RPL10 |  |  | 53 | 0.344 | 0.0756 | Yes |
| 4 | HES6 |  |  | 60 | 0.336 | 0.1002 | Yes |
| 5 | SOX4 |  |  | 91 | 0.298 | 0.1029 | Yes |
| 6 | MARCKS |  |  | 96 | 0.295 | 0.1254 | Yes |
| 7 | TMEM158 |  |  | 120 | 0.275 | 0.1315 | Yes |
| 8 | MEF2A |  |  | 163 | -0.251 | 0.1207 | No |
| 9 | TAGLN3 |  |  | 257 | -0.260 | 0.0712 | No |
| 10 | THSD7A |  |  | 268 | -0.261 | 0.0862 | No |
| 11 | SORBS2 |  |  | 318 | -0.265 | 0.0712 | No |
| 12 | TUBB |  |  | 410 | -0.274 | 0.0245 | No |
| 13 | NREP |  |  | 509 | -0.286 | -0.0265 | No |
| 14 | LZTS1 |  |  | 564 | -0.293 | -0.0430 | No |
| 15 | AUTS2 |  |  | 590 | -0.297 | -0.0365 | No |
| 16 | MEIS2 |  |  | 594 | -0.298 | -0.0129 | No |
| 17 | ENC1 |  |  | 643 | -0.309 | -0.0233 | No |
| 18 | USP48 |  |  | 686 | -0.316 | -0.0283 | No |
| 19 | TRIM37 |  |  | 689 | -0.317 | -0.0023 | No |
| 20 | TMSB4X |  |  | 742 | -0.329 | -0.0140 | No |
| 21 | IDS |  |  | 782 | -0.339 | -0.0148 | No |
| 22 | CASK |  |  | 832 | -0.353 | -0.0221 | No |
| 23 | RND3 |  |  | 853 | -0.358 | -0.0065 | No |
| 24 | XPO1 |  |  | 967 | -0.400 | -0.0592 | No |
| 25 | CEP78 |  |  | 984 | -0.408 | -0.0362 | No |
| 26 | ROBO2 |  |  | 1037 | -0.438 | -0.0384 | No |
| 27 | ITPR2 |  |  | 1053 | -0.447 | -0.0112 | No |
| 28 | NTM |  |  | 1094 | -0.466 | -0.0016 | No |
| 29 | GRIA2 |  |  | 1164 | -0.526 | -0.0094 | No |
| 30 | PDE1C |  |  | 1232 | -0.651 | -0.0047 | No |
| 31 | SYNE2 |  |  | 1282 | -0.829 | 0.0295 | No |
Table: GSEA details [plain text format]

  

Fig 2: RICHARDS\_NOWAKOWSKI\_SCIENCE\_2017\_IPC\_NEN1\_UPREG\_1: Random ES distribution      
 Gene set null distribution of ES for **RICHARDS\_NOWAKOWSKI\_SCIENCE\_2017\_IPC\_NEN1\_UPREG\_1**
